# Supplementary material for: ROS homeostasis mediated by MPK4 and SUMM2 determines synergid cell death
Source: Nat Commun. 2022 Apr 1;13:1746. doi: 10.1038/s41467-022-29373-7 (PMC8976062; doi:10.1038/s41467-022-29373-7)
Supplement: Supplementary file 1 — Supplementary Information [file 41467_2022_29373_MOESM1_ESM.pdf]

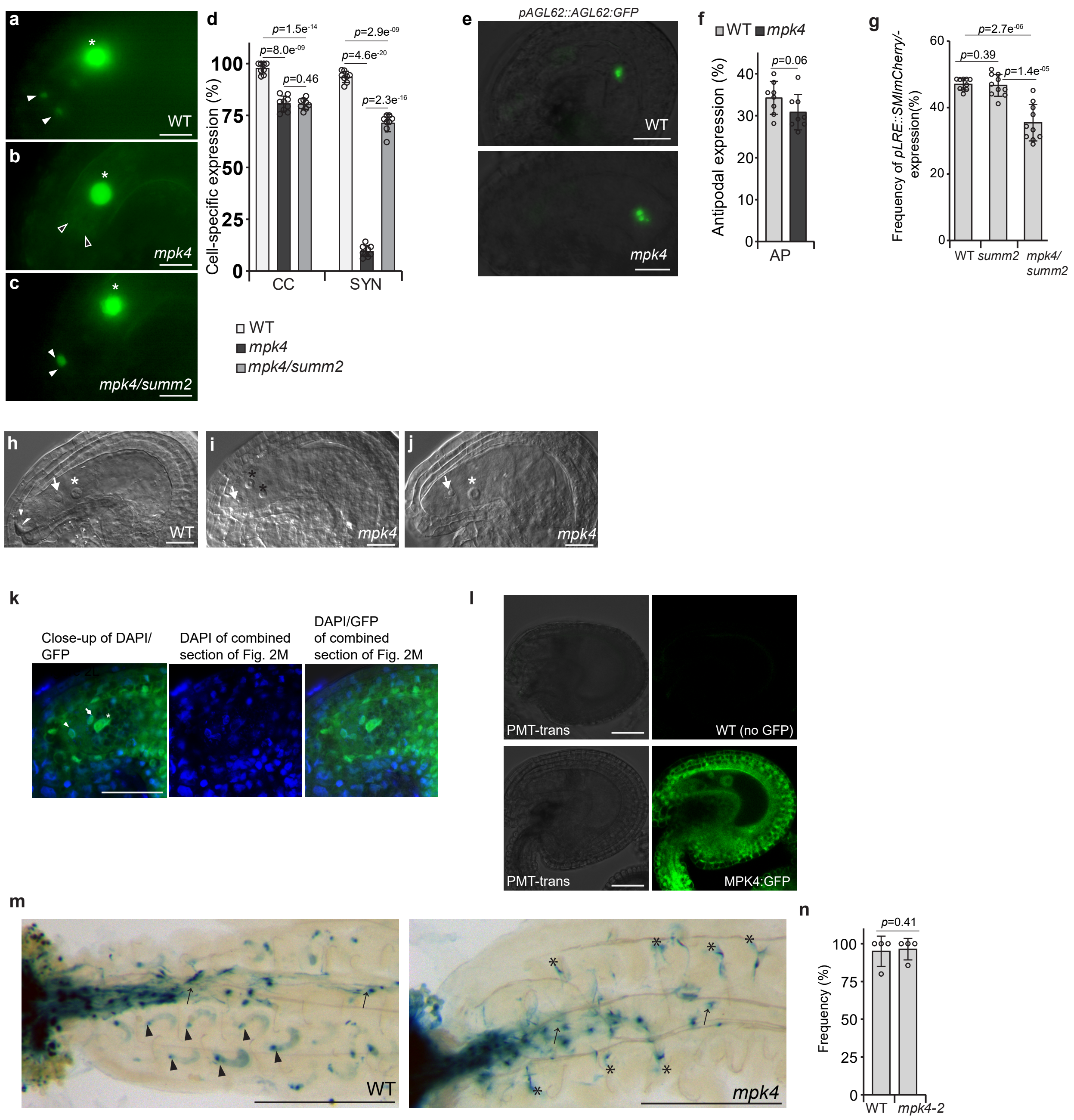


Supplementary Figure 1

**Supplementary Figure 1. Characterization of the premature synergid degeneration and the MPK4:GFP localization**

**a-d** Localization (a-c) and quantification (d) of the female gametophyte reporter *pHD2B::GFP:HD2B/+* in WT (n=263), *mpk4* (n=310) and *mpk4/summ2* (n=283). Error bars show ± SD.
**e-f** Localization and occurrence of the antipodal marker *pAGL62::AGL62:GFP/-* (WT, n=249; *mpk4*, n=195). Error bars show ± SD. White arrowhead, synergid; black arrowhead, degenerating synergid; white arrow, egg cell; white asterisk, central cell nucleus; Scale bars, 20 µm
**g** Frequency of *pLRE::SMImCherry/-* expression in WT (n=338), *summ2* (n=332) and *mpk4/summ2* (n=314). See also **Figure 4o-p**. Error bars show ± SD.
**h-j** Differential-contrast microscopy of WT (h) and *mpk4* (i-j) female gametophyte. Representative images are depicted of three biological replicates. Scale bars, 20 µm.
**k** MPK4:GFP localization in synergid, egg cell, CC and sporophytic ovule tissue, co-localized with the nuclei staining dye 4′,6-diamidino-2-phenylindole DAPI, **also see Figure 2n**. Scale bars, 20 µm
**l** Evaluation of MPK4:GFP signal intensity against a WT control without carrying a GFP-reporter.
**m-n** GUS-stained PT tracking through the transmitting tract in WT and *mpk4*. Flowers of WT and *mpk4* were pollinated with *pLAT52::GUS*-expressing pollen and the growth of the PT within the maternal tissue was evaluated after 24 hours. Images show regular growth of the PT in the transmitting tract/ pistil in WT and *mpk4*, yet, ovule-targeting in *mpk4* is affected (Black asterisk). Black arrowhead, PT-arrival and burst in the FG; black arrow, PT in the transmitting tract of the pistil. Shown are the results of 4 biological replicates. Error bars show ± SD.
Statistical significance was analyzed by one-way ANOVA. Source data and further statistical analysis are provided in the source data file.

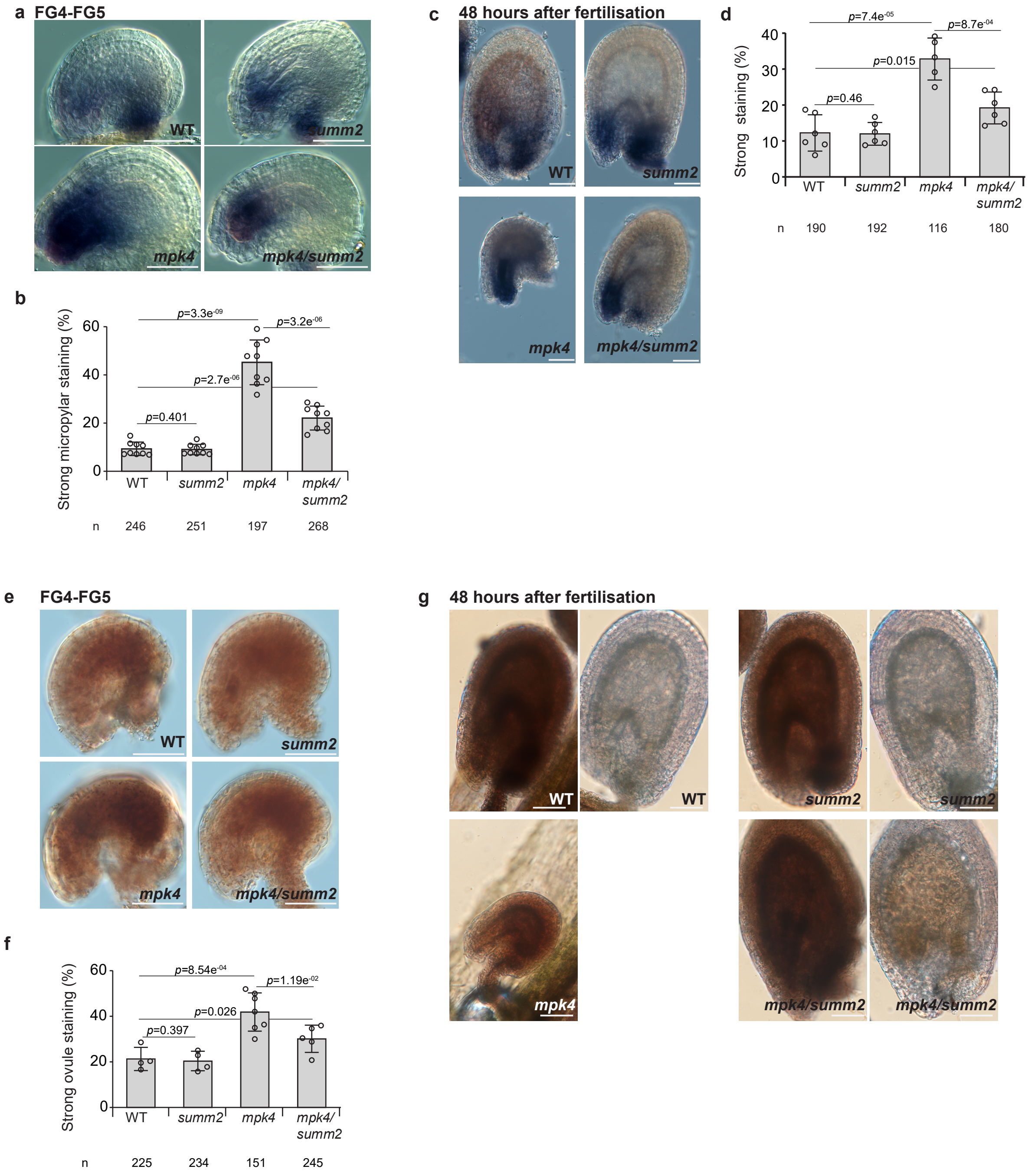


Supplementary Figure 2

**Supplementary Figure 2. Analysis of reactive-oxygen species levels before and after fertilization**
**a-g** NBT-staining (a-d) and DAB staining (e-g) at the FG4-FG5 developmental stage and 48 hours after fertilization. Depicted are the results and representative images of three biological replicates. Statistical significance was analyzed by one-way ANOVA. Error bars show ± SD. Source data and further statistical analysis are provided in the source data file. Scale bars, 30 µm.

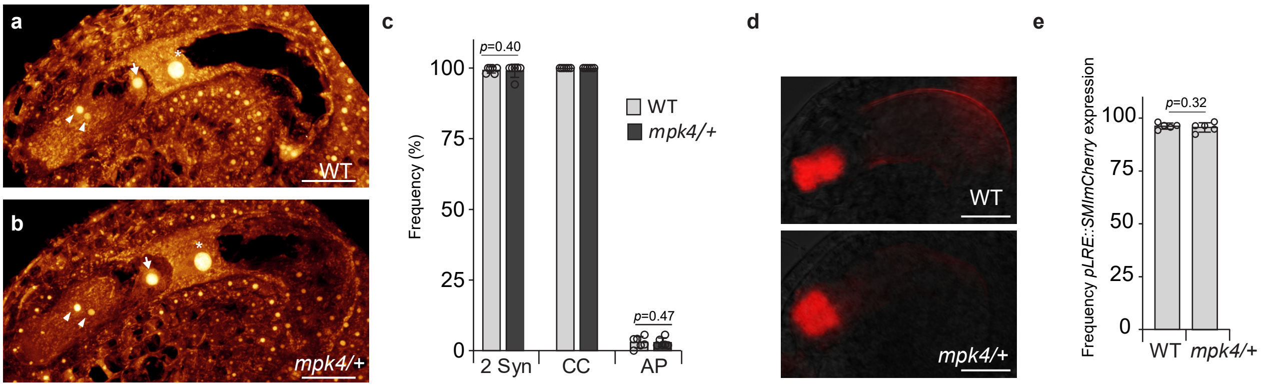


**f** *mpk4-2* transmission efficiency

| | | determined | | | expected | | |
|---|---|---|---|---|---|---|---|
| **Transmission** | **cross** | **+/+** | ***mpk4-2/+*** | ***mpk4-2/mpk4-2*** | **+/+ (%)** | ***mpk4-2/+* (%)** | ***mpk4-2/mpk4-2*** |
| selfed | -/+ x -/+ | 91 (28.4%) | 171 (53.5%) | 58 (18.1%) | 25.0 | 50.0 | 25.0 |

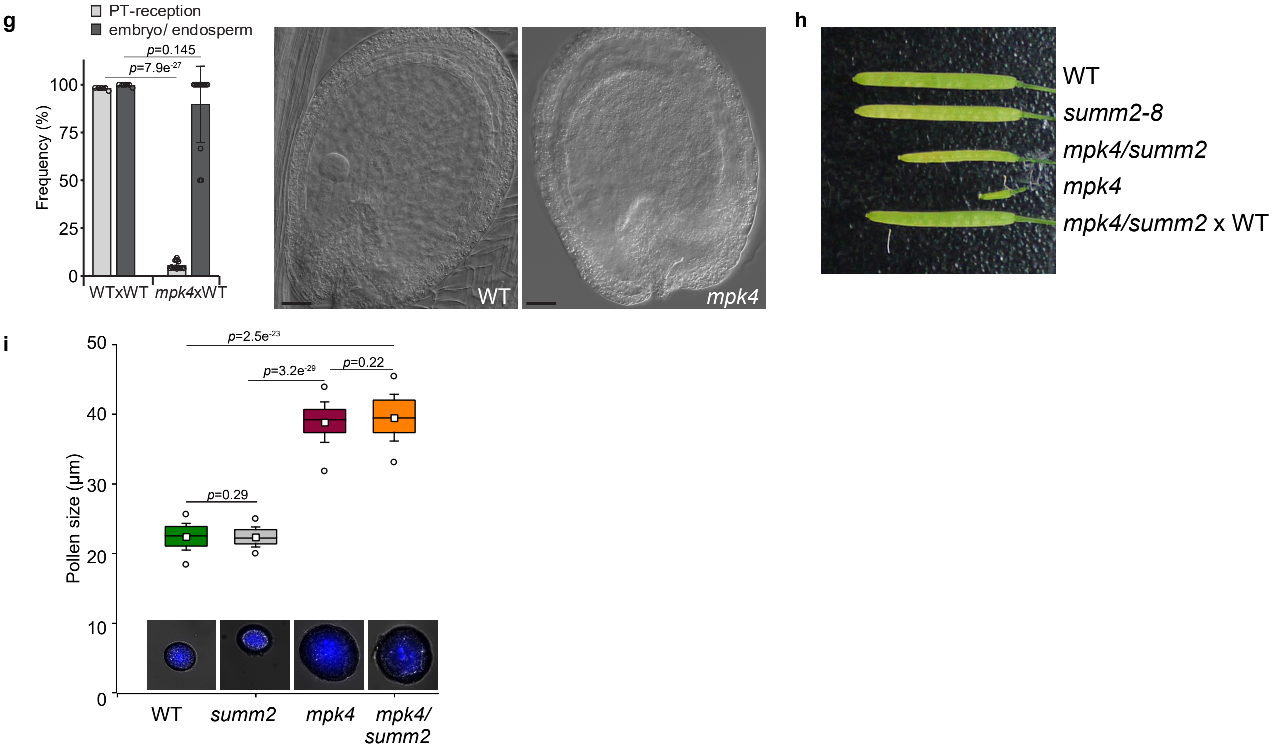


Supplementary Figure 3

**Supplementary Figure 3. Characterization of the heterozygote *mpk4* mutant**

**a-c** The occurrence of synergids, central cell and antipodals in WT (n=298) and *mpk4/+* (n=304), see also **Supplementary movies 7-8**. Depicted are the results and representative images of three biological replicates. Error bars show ± SD. Scale bars, 30 µm.

**d-e** The expression of the *pLRE::SM1-mCherryGolgie* reporter in the synergids in WT (n=268) and *mpk4/+* (n-279). Depicted are the results and representative images of three biological replicates. Error bars show ± SD. Scale bars, 20 µm.

**f** Transmission efficiency of the self-crossed heterozygote *mpk4* mutant.

**g** Frequency of embryo/endosperm formation in WT (n=302) and *mpk4/-* (n=304) after the reception of WT-pollen tubes. Three biological replicates analyzed. Error bars show ± SD. Scale bars, 30 µm.

**h** Representative siliques of WT, *summ2-8*, *mpk4/summ2*, *mpk4*, and *mpk4/summ2* pollinated with WT pollen.

**i** Pollen size of WT, *summ2*, *mpk4/summ2,* and *mpk4*. Boxes represent the 25th and 75th percentiles, and the inner rectangle highlights the median, whiskers show the SD, and outliers are depicted by dots (Min/max range). Statistical significance was analyzed by one-way ANOVA. Analyzed were three biological replicates. Source data and further statistical analysis are provided in the source data file.

Supplementary Table 1

| **Primer used in this study:** | | |
|---|---|---|
| mpk4-LP | GTGACAATGCAAGAAGATACGTTAGACAGC | Genotyping *mpk4-2* |
| mpk4-RP | CTTGAAATATCTACAGAGTTGGTGTG | Genotyping *mpk4-2* |
| LBb1 | ATTTTGCCGATTTCGGAAC | T-DNA, genotyping *mpk4-2* |
| 91s | TTTCTGTATCAGTTGTTGCGTG | Genotyping *mpk4-3* , base pair exchange determination by sequencing: GaGGATTTCGGG |
| 91as | TCTAACGTATCTTCTTGCATTGTC | Genotyping *mpk4-3* , GaGGATTTCGGG |
| LB3 | atctgaatttcataaccaatctcgatacac | T-DNA, genotyping *summ2-8* |
| 90s | ATGGGAGCTTGTTTAACACTCTCG | Genotyping *summ2-8* |
| 87as | CCAATGGAAGCTTTCTCAGC | Genotyping *summ2-8* |
