# Supplementary material for: ROS homeostasis mediated by MPK4 and SUMM2 determines synergid cell death
Source: Nat Commun. 2022 Apr 1;13:1746. doi: 10.1038/s41467-022-29373-7 (PMC8976062; doi:10.1038/s41467-022-29373-7)
Supplement: Supplementary file 3 — Description of Additional Supplementary Files [file 41467_2022_29373_MOESM3_ESM.pdf]

Description of Additional Supplementary Files

Animated 3D-projections of CLSM Z-stacks of WT, *mpk4* and *mpk4/summ2* female gametophytes, with a focal plane distance of 1-2 µm within a total range of about 20-30 µm.

File name: Supplementary Movie 1

Description: associated with Figure 1i_WT

Title: Mature WT female gametophyte

File name: Supplementary Movie 2

Description: associated with Figure 1k_*mpk4*

Title: Mature *mpk4* female gametophyte showing synergids that undergo premature degeneration.

File name: Supplementary Movie 3

Description: associated with Figure 1m_*mpk4*

Title: Mature *mpk4* female gametophyte showing prematurely degenerating synergids

File name: Supplementary Movie 4

Description: associated with Figure 1o_*mpk4*

Title: Mature *mpk4* female gametophyte with entirely degenerated synergids

File name: Supplementary Movie 5

Description: associated with Figure 2l_MPK4:GFP

Title: MPK4:GFP localization in the micropylar ovule region in WT background

File name: Supplementary Movie 6

Description: associated with Figure 4d_*mpk4/summ2*

Title: Mature *mpk4/summ2* female gametophyte with restored synergid formation.

File name: Supplementary Movie 7

Description: associated with Supplementary Figure 3a_WT

Title: WT female gametophyte

File name: Supplementary Movie 8

Description: associated with Supplementary Figure 3b_*mpk4/+*

Title: Female gametophyte of a heterozygote *mpk4* plant
